# Epidemiology of *Taenia saginata* taeniosis/cysticercosis: a systematic review of the distribution in the Americas

**DOI:** 10.1186/s13071-018-3079-y

**Published:** 2018-09-20

**Authors:** Uffe Christian Braae, Lian F. Thomas, Lucy J. Robertson, Veronique Dermauw, Pierre Dorny, Arve Lee Willingham, Anastasios Saratsis, Brecht Devleesschauwer

**Affiliations:** 1 One Health Center for Zoonoses and Tropical Veterinary Medicine, Ross University School of Veterinary Medicine, P.O. Box 334, Basseterre, Saint Kitts and Nevis; 2 International Livestock Research Institute (ILRI), P.O. Box 30709, Nairobi, Kenya; 3 Institute for Infection & Global Health, University of Liverpool, Neston, UK; 4 Parasitology, Department of Food Safety and Infection Biology, Faculty of Veterinary Medicine, Norwegian University of Life Sciences, Adamstuen Campus, Oslo, Norway; 5 Department of Biomedical Sciences, Institute of Tropical Medicine, Antwerp, Belgium; 6 Department of Virology, Parasitology and Immunology, Faculty of Veterinary Medicine, Ghent University, Merelbeke, Belgium; 7 Veterinary Research Institute, Hellenic Agricultural Organization Demeter, 57001 Thermi, Greece; 8 Department of Epidemiology and Public Health, Sciensano, Brussels, Belgium; 9 Department of Veterinary Public Health and Food Safety, Faculty of Veterinary Medicine, Ghent University, Merelbeke, Belgium

**Keywords:** *Taenia saginata*, Cestoda, Beef tapeworm, Taeniosis, Bovine cysticercosis, North and South America, Caribbean

## Abstract

**Background:**

The distribution of *Taenia saginata* in the Americas is unclear. Establishing the distribution, economic burden, and potentials for control of bovine cysticercosis is increasingly important due to the growing demand for beef. This paper aims to take the first step and reviews the recent distribution of *T. saginata* taeniosis and bovine cysticercosis on a national level within the Americas.

**Methods:**

We undertook a systematic review of published and grey literature for information on the occurrence, prevalence, and geographical distribution of bovine cysticercosis and human taeniosis in the 54 countries and territories of the Americas between January 1st, 1990 and December 31st, 2017. Data on bovine cysticercosis from OIE reports from 1994 to 2005 were also included.

**Results:**

We identified 66 papers from the Americas with data on the occurrence of taeniosis or bovine cysticercosis and an additional 19 OIE country reports on bovine cysticercosis. Taeniosis was reported from 13 countries, with nine of these countries reporting specifically *T. saginata* taeniosis, and four countries reporting non-species specific taeniosis. The reported prevalence of taeniosis ranged between 0.04–8.8%. Bovine cysticercosis was reported from 19 countries, nine identified through the literature search, and an additional 10 identified through the OIE country reports for notifiable diseases. The reported prevalence of bovine cysticercosis ranged between 0.1–19%. Disease occurrence was restricted to 21 countries within the Americas, the majority from the mainland, with the only island nations reporting either bovine cysticercosis or taeniosis being Cuba, Haiti, and the US Virgin Islands.

**Conclusions:**

*Taenia saginata* is widely distributed across 21 of the 54 countries in the Americas, but insufficient epidemiological data are available to estimate the subnational spatial distribution, prevalence, incidence and intensity of infections. This needs to be addressed through active surveillance and disease detection programmes. Such programmes would improve the data quantity and quality, and may enable estimation of the economic burden due to bovine cysticercosis in the region in turn determining the requirement for and cost-effectiveness of control measures.

**Electronic supplementary material:**

The online version of this article (10.1186/s13071-018-3079-y) contains supplementary material, which is available to authorized users.

## Background

*Taenia saginata* is a zoonotic tapeworm that is of economic importance in countries where cattle are kept. The parasite is transmitted from human tapeworm carriers (taeniosis) to bovines (cysticercosis) by excretion of eggs or proglottids containing eggs into the environment *via* the stool. Bovines can then ingest the eggs through contaminated feed or water. After ingestion, the eggs hatch and release oncospheres in the small intestines, where the oncospheres penetrate the intestinal wall to reach the blood circulation. This distributes them throughout the body, but primarily to muscle tissue, where they develop into cysticerci. For humans to become infected with *T. saginata*, raw or undercooked bovine meat or offal containing infective cysts must be consumed. Bovine cysticercosis has been associated with various environmental factors related to water sources, such as animals having access to surface water, flooding of pastures and proximity to wastewater sources [1].

Taeniosis causes only a few, if any, mild symptoms in humans [2], and bovine cysticercosis is usually asymptomatic. The primary burden of the parasite is therefore the economic burden imposed on the cattle industry. Economic losses occur when infected carcasses are identified during routine meat inspection at slaughter facilities, causing total economic loss if the carcass is condemned due to high intensity infection, or partial economic loss if extra-processing of the carcass is required due to low intensity infection. Additional costs may include increased labour costs due to extra-handling and transport of infected carcasses to appropriate facilities, in addition to, potential freezing, transport and processing of the meat. However, the current economic burden due to bovine cysticercosis in the Americas has not been estimated.

*Post-mortem* inspection procedures of carcasses for pathogens vary from country to country, and even from facility to facility in some countries. In general, however, this diagnostic method has low sensitivity for detection of bovine cysticercosis [3–5]. Nevertheless, routine meat inspection remains the preferred tool for *T. saginata* detection in bovines. There is currently no *ante-mortem* test that performs with high sensitivity and high specificity, regardless of infection intensity. The sensitivities of existing serological tests are highly dependent on the infection intensity within the host [6], with the tests becoming increasingly unreliable as infection intensity decreases. The lack of a ‘gold standard’, combined with the non-specific symptomatic/asymptomatic nature of the diseases caused by the parasite in humans and bovines, and the prolonged survival of *T. saginata* eggs in the environment [7], makes *T. saginata* difficult to control.

*Taenia saginata* is thought to be widely distributed throughout the world, and to a larger degree in low-income countries where hygiene and sanitation standards are below average and routine meat inspection not always enforced. Nonetheless, in countries where standards of hygiene and sanitation are considered high and routine meat inspection enforced, such as within Europe, bovine cysticercosis still remains widely distributed [8]. There is no clear overview of the distribution of this zoonotic cestode in the Americas, and with a growing demand for beef, establishing the distribution, prevalence, economic burden, and potentials for control is more important than ever. This paper aims to take the first step, and reviews the distribution of *T. saginata* taeniosis and bovine cysticercosis on a national level within the Americas between 1990 and 2017.

## Methods

### Search strategy

We undertook a systematic review of published literature for information on the occurrence, prevalence, and geographical distribution of bovine cysticercosis and human taeniosis in the Americas between January 1st, 1990 and December 31st, 2017, using an approach that followed PRISMA guidelines [9]. The protocol and the PRISMA checklist for this review can be found in Additional file 1. The Americas for the purpose of this review included the following 54 countries or territories: Anguilla, Antigua & Barbuda, Argentina, Aruba, Bahamas, Barbados, Belize, Bermuda, Bolivia, Brazil, British Virgin Islands, Canada, Caribbean Netherlands, Cayman Islands, Chile, Colombia, Costa Rica, Cuba, Curacao, Dominica, Dominican Republic, Ecuador, El Salvador, Falkland Islands, French Guiana, Greenland, Grenada, Guadeloupe, Guatemala, Guyana, Haiti, Honduras, Jamaica, Martinique, Mexico, Montserrat, Nicaragua, Panama, Paraguay, Peru, Puerto Rico, Saint Barthélemy, Saint Kitts & Nevis, Saint Lucia, Saint Martin, Saint Pierre & Miquelon, Saint Vincent & the Grenadines, Suriname, Trinidad & Tobago, Turks & Caicos Islands, Uruguay, US Virgin Islands, USA and Venezuela.

The first search was done in PubMed (http://www.ncbi.nlm.nih.gov/pubmed), using the following search phrase: (cysticerc* OR cisticerc* OR "C. bovis" OR taenia* OR tenia* OR saginata OR taeniosis OR teniosis OR taeniasis OR ténia OR taeniid OR cysticerque OR Taeniarhynchus) AND (America OR USA OR Brazil OR Argentina OR Canada OR Peru OR Chile OR Ecuador OR Bolivia OR Paraguay OR Costa Rica OR Uruguay OR Bermuda OR Greenland OR Caribbean Netherlands OR Saint Barts OR Saint Pierre and Miquelon OR Falkland Islands OR Anguilla OR Antigua and Barbuda OR Aruba OR Bahamas OR Barbados OR Belize OR Bonaire OR British Virgin Islands OR Bermuda OR Cayman Islands OR Colombia OR Costa Rica OR Cuba OR Curaçao OR Dominica OR Dominican Republic OR El Salvador OR French Guiana OR Grenada OR Guadeloupe OR Guatemala OR Guyana OR Haiti OR Honduras OR Jamaica OR Martinique OR Mexico OR Montserrat OR Netherlands Antilles OR Nicaragua OR Panama OR Puerto Rico OR Saba OR Saint Kitts and Nevis OR Saint Lucia OR Saint Vincent and the Grenadines OR Saint Eustatius OR Sint Maarten OR Saint Martin OR Suriname OR Trinidad and Tobago OR Turks and Caicos Islands OR US Virgin Islands OR Venezuela). The following databases were also searched using keywords from the above search phrase: Web of Science (www.webofknowledge.com), OpenGrey (http://www.opengrey.eu/), and CABDirect (http://www.cabdirect.org/).

### Selection criteria

**Fig. 1:**
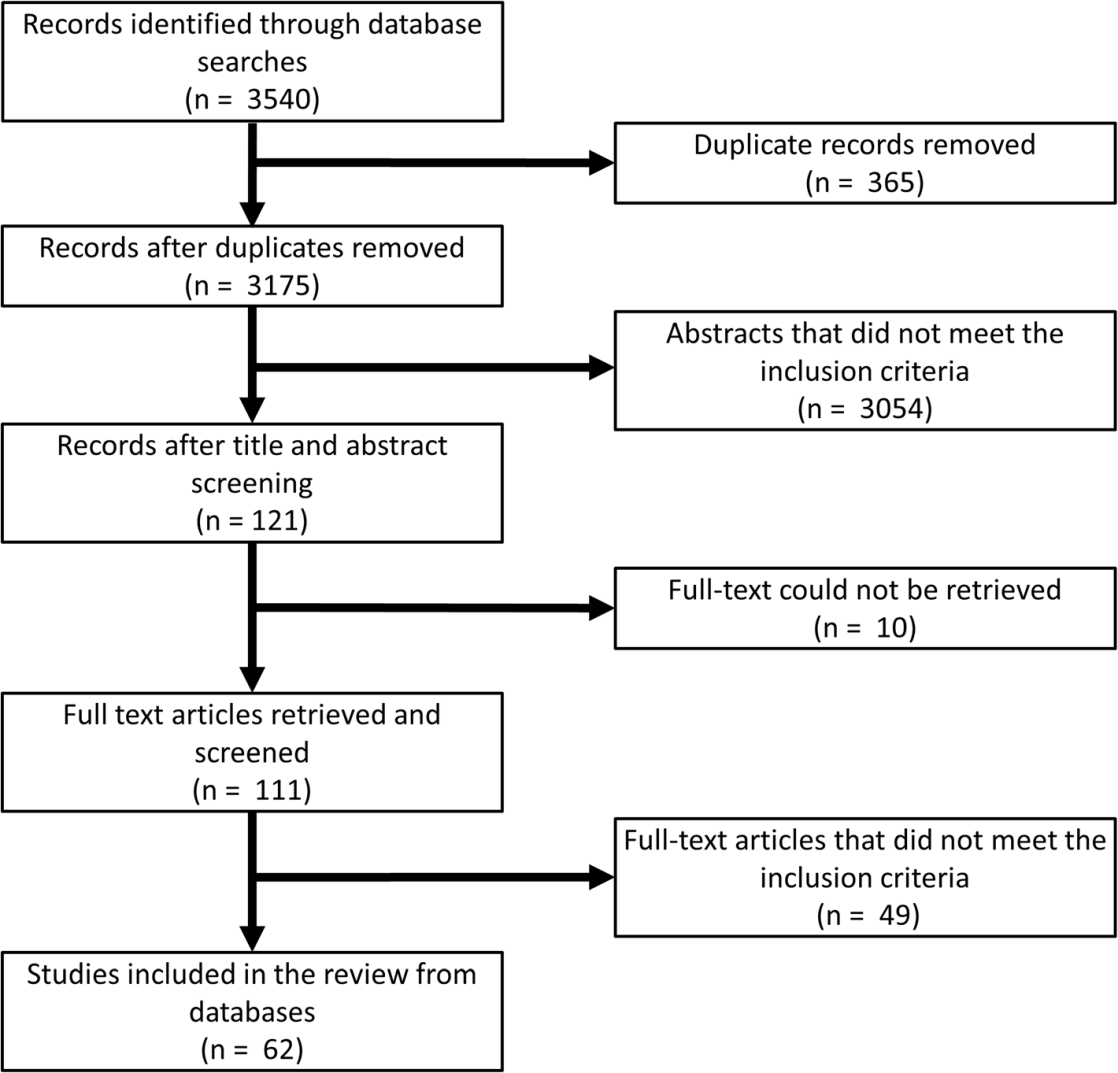
Flow diagram of the database searches

Outputs from the databases searches were compiled and screened for duplicates. Thereafter, titles and abstracts were screened for eligibility and were excluded on the following grounds: (i) studies concerning a parasite other than *T. saginata*; (ii) studies reporting data from countries different from those listed above; (iii) studies published prior to January 1st, 1990 or after December 31st, 2017; (iv) studies reporting results outside the scope of the review question (e.g. laboratory experiments, environmental studies and general reviews); and (v) duplicated data. If the same data had been published more than once, the oldest article was included and all others omitted. Full text manuscripts were then retrieved where possible and assessed by the same criteria as above (Fig. 1). The citations in identified reports were also screened for relevant literature.

Data on bovine cysticercosis from OIE reports from 1994 to 2005 were also obtained [10, 11]. Additionally, we sought to obtain data from known articles not captured in the literature database searches, as well as unpublished work (i.e. masters’ theses) and these were included if they confirmed presence of *T. saginata* from a country where no disease report had been identified in the literature search, or if prevalence data were presented at a higher geographical resolution to that in the published literature.

### Data extraction and generation

From the included literature and reports, data were extracted into predefined tables that can be found in Additional file 2: Tables S1-S3. Prevalence data were only extracted if both the numerator and the denominator were provided, and 95% confidence intervals using the Clopper-Pearson method, were calculated, if not already stated within the paper. All maps were generated using ArcGIS 10.3.1 (ESRI Inc., USA).

## Results

### Search results

The database searches yielded 62 articles presenting data on taeniosis or bovine cysticercosis in the Americas. An additional four articles were identified from other sources and confirmed occurrence of taeniosis in Venezuela, and occurrence of bovine cysticercosis in the USA and the US Virgin Islands. Of the 66 articles identified, 31 reported occurrences of taeniosis, 33 reported occurrences of bovine cysticercosis, and two papers reported occurrences of both diseases. A total of 19 OIE country reports were also identified. All eligible references are listed within the tables of this paper.

### Taeniosis and bovine cysticercosis occurrence

In the period 1990–2017, taeniosis or bovine cysticercosis has been reported within all mainland countries in the Americas except for Belize, French Guiana, Guyana, Panama and Suriname. The only island nations within the region to report any disease occurrence during the study period were Cuba, Haiti and the US Virgin Islands, contributing to occurrence of the parasite in a total of 21 countries within the Americas.

### Human taeniosis occurrence

**Fig. 2:**
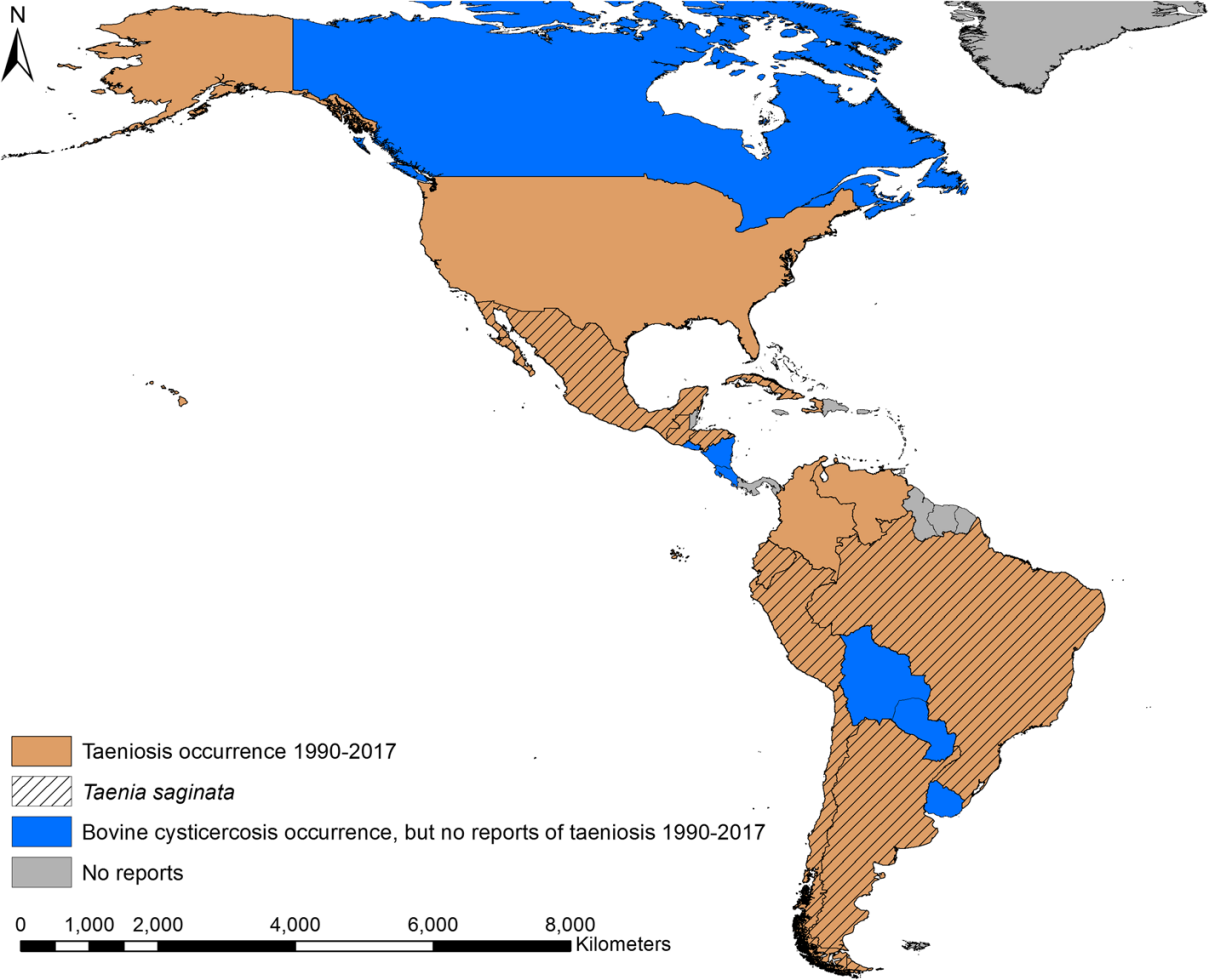
Countries with reports of taeniosis due to *Taenia saginata* and *Taenia* spp. in the period 1990–2017

In the period 1990–2017, taeniosis was reported in 13 countries in the Americas, with *T. saginata* taeniosis reported in nine of those countries (Fig. 2). Colombia, Haiti, Venezuela and the USA reported taeniosis, but the specific tapeworm species were not confirmed in any of the reports. In seven countries there were reports of bovine cysticercosis, but no reports of taeniosis. In total, 33 papers reporting the occurrence of taeniosis within the Americas were identified in the search strategy. Of the 33 papers, there were three case reports of taeniosis, with species identification done in two of these cases, Chile and Mexico (Table 1). In the case reports from Brazil and Chile, it was unclear exactly when the infection had been discovered.

**Table 1:** Individual cases of human taeniosis (published case studies)

Country	Year	Nationality	Species^a^	Diagnostic technique	Reference
Brazil	na	Brazilian	*Taenia* spp.	Proglottid identification	[27]
Chile	na	na	*T. saginata*	Proglottid identification	[28]
Mexico	2006	na	*T. saginata*	Proglottid identification	[29]

*Abbreviation*: *na* information not provided

^a^If confirmed

Of the 33 papers, seven contained insufficient data, or the data were of insufficient quality to yield prevalence estimations (Table 2). In the majority of these studies, species identification was performed, but the diagnostic method was not always described.

**Table 2:** Aggregated cases of human taeniosis (hospital/laboratory/field records without prevalence data)

Country	Year	No. of cases	Species^a^	Diagnostic technique	Reference
Brazil	na	na	*Taenia* spp.	Ritchie technique	[30]
Colombia	2009–2013	na	*T. saginata*	na	[31]
Honduras	na	4	*T. saginata*	Worm expulsion	[32]
Peru	2004–2007	16	*T. saginata*	Worm expulsion	[33]
Peru	1998–2000	11	*T. saginata*	Worm expulsion	[34]
Venezuela	na	1	*Taenia* spp.	Ritchie technique	[35]
Venezuela	2004	18	*Taenia* spp.	na	[36]

*Abbreviation*: *na* information not provided

^a^If confirmed

In 23 papers, the methodology and data were sufficiently described to enable prevalence and confidence interval calculations (Table 3). In the majority, disease confirmation was based on various microscopic methods. Prevalence reports ranged from between 0.04–8.8%. All 23 studies reported data that could be georeferenced to first-level administration.

**Table 3:** Prevalence of human taeniosis (published data)

Country	Year	Location of study^a^	Time frame	Prevalence (%) (95% CI)	Species	Diagnostic technique	Reference
Argentina	1993	Corrientes	Apr-Oct	0.48 (0.01–2.66)	*T. saginata* ^b^	Microscopy	[37]
Argentina	2005	Corrientes	Mar-Dec	1.77 (0.22–6.25)	*Taenia* spp.	Graham tests	[38]
Brazil	na	Minas Gerais	na	0.18 (0.13–0.26)	*Taenia* spp.	Kato-Katz	[39]
Brazil	2000–2001	Minas Gerais	na	0.04 (0.03–0.05)	*T. saginata*	Worm expulsion	[40]
Brazil	na	Mato Grosso do Sul	na	2.24 (0.46–6.40)	*Taenia* spp.	Microscopy	[41]
Brazil	2004–2006	Paraná	Jun-May	0.23 (0.00–0.28)	*Taenia* spp.	Microscopy	[42]
Brazil	1992–1993	Minas Gerais	Jan-Dec	2.22 (1.43–3.29)	*Taenia* spp.	Microscopy	[43]
Brazil	1992	Minas Gerais	na	0.72 (0.46–1.07)	*Taenia* spp.	Microscopy	[44]
Chile	2005–2008	Maule	Jan-Dec	0.13 (0.07–0.21)	*Taenia* spp.	Microscopy	[45]
Colombia	2004	Bolívar	Feb-Jun	0.79 (0.16–2.28)	*Taenia* spp.	Microscopy	[46]
Ecuador	2000	Imbabura	Jan-May	0.40 (0.13–0.94)	*T. saginata*	Ritchie technique & worm expulsion	[47]
		Pichincha		2.30 (1.32–3.71)			
Guatemala	na	Jutiapa	na	0.06 (0.010.33)	*T. saginata*	Worm expulsion	[48]
Guatemala	1991–1995	Jutiapa	Oct-Jan	0.03 (0.01–0.16)	*T. saginata* ^b^	Copro-antigen ELISA	[49]
Haiti	2002	Nationwide	na	0.31 (0.18–0.49)	*Taenia* spp.	Ritchie technique	[50]
Honduras	na	Francisco Morazán	na	0.25 (0.01–1.37)	*Taenia* spp.	Ritchie technique	[51]
Mexico	2004	Chihuahua	Aug-Dec	1.02 (0.21–2.96)	*Taenia* spp.	Microscopy & Copro-antigen ELISA	[52]
Mexico	1992	Baja California	Feb-Jul	6.5 (2.43–13.66)	*Taenia* spp.	Microscopy	[53]
Mexico	1998	Guerrero	na	0.74 (0.15–2.16)	*Taenia* spp.	Copro-antigen ELISA	[54]
Mexico	1996	Morelos	na	0.50 (0.10–1.44)	*Taenia* spp.	Copro-antigen ELISA & Ritchie technique	[55]
Peru	2008	Ayacucho	na	1.4 (0.44–3.14)	*Taenia* spp.	Microscopy	[56]
Peru	na	Tumbes	na	1.5 (0.55–3.22)	*Taenia* spp.	Copro-antigen ELISA	[57]
USA	2004	Texas	Aug-Dec	8.8 (4.11–16.09)	*Taenia* spp.	Microscopy & Copro-antigen ELISA	[52]
Venezuela	na	Tachira	na	0.8 (0.02–4.31)	*Taenia* spp.	Ritchie technique	[58]

*Abbreviations*: *CI* confidence interval, *na* information not provided

^a^First-level administration

^b^No description of species confirmation

### Bovine cysticercosis

**Fig. 3:**
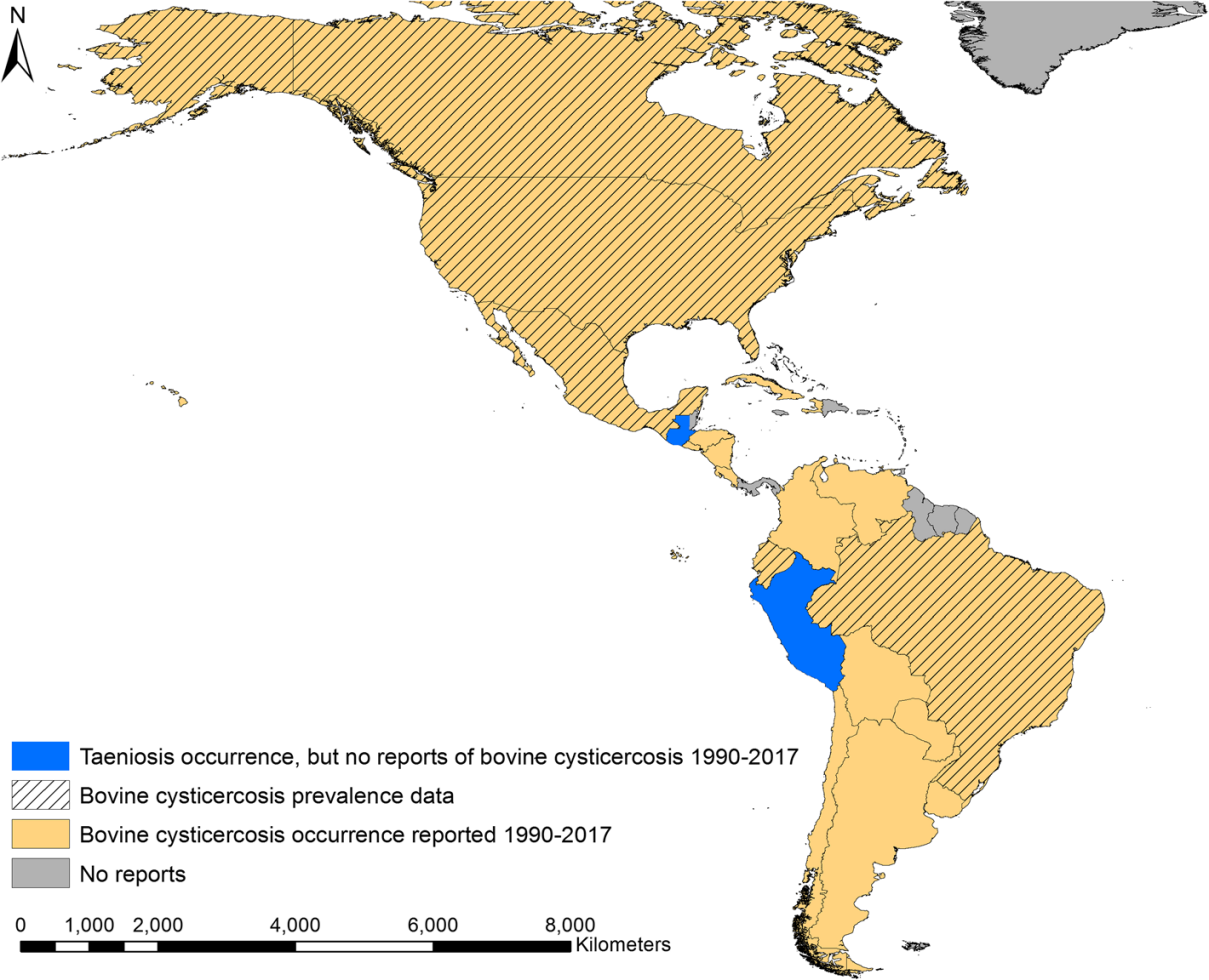
Bovine cysticercosis occurrence and countries with studies reporting prevalence in the period 1990–2017

Bovine cysticercosis was reported from 19 countries within the Americas during the period 1990–2017. The literature search identified nine countries with bovine cysticercosis, and an additional 10 countries were identified through the 1994 and 2005 OIE country reports for notifiable diseases [10, 11]. *Taenia saginata* was reported from humans in both Guatemala and Peru during 1990–2017, but no reports of bovine cysticercosis could be obtained from these two countries (Fig. 3). Cuba, Haiti and the US Virgin Islands were the only island nations/territories to report bovine cysticercosis during the study period. On the mainland most countries reported bovine cysticercosis, but no reports could be found from Belize, French Guiana, Guyana, Suriname and Panama. Of the 35 papers identified that reported occurrence of bovine cysticercosis, seven did not contain sufficient data for prevalence calculations. All seven papers reported results of official meat inspections in Brazil, Chile, Cuba, the USA and the US Virgin Islands, respectively (Table 4).

**Table 4:** Reported occurrence of bovine cysticercosis (case studies/published data without full prevalence or incidence data) based on meat inspection

Country	Year	Location of study^a^	Cases	Reference
Brazil	na	Minas Gerais	na	[59]
Brazil	2005–2006	Goias	na	[60]
Brazil	2009–2010	Minas Gerais	2019	[61]
Brazil	2008–2010	Espírito Santo	na	[62]
Chile	2010	na	148	[63]
Cuba	1998–2001	Villa Clara	na	[64]
USA	1985–1994	Nationwide	na	[23]
US Virgin Islands	1992	na	na	[23]

*Abbreviation*: *na* information not provided

^a^First-level administration

In total, 28 papers contained sufficient information to calculate prevalence and 95% confidence intervals (Table 5). Most of the studies (82%) originated from Brazil, where reported prevalence ranged between 0.1–19%, with both ends of this range obtained by routine meat inspection. Overall, the majority of the studies reported official meat inspection data, but antibody detection (by Ab-ELISA or immunoblot, studies in Brazil) and antigen detection (Ag-ELISA, study in Ecuador) were also used as diagnostic techniques.

**Table 5:** Prevalence of bovine cysticercosis (published data)

Country	Year	Location of study^a^	Time frame	Prevalence (95% CI)	Diagnostic technique	Reference
Brazil	2013	Minas Gerais	Jan-Jun	2.1 (2.01–2.22)	Meat inspection	[65]
Brazil	2006–2007	Bahia	na	0.7 (0.64–0.67)	Meat inspection	[66]
Brazil	2004	Minas Gerais	na	3.2 (3.00–3.48)	Meat inspection	[67]
Brazil	na	Mato Grosso do Sul	na	18.8 (11.51–28.00)	Meat inspection	[68]
Brazil	2000	Paraná	Jul-Dec	3.8 (3.60–4.07)	Meat inspection	[69]
Brazil	2013	Minas Gerais	Jul-Aug	15.1 (12.91–17.44)	Ab-ELISA	[70]
				4.7 (3.47–6.19)	Immunoblot	
Brazil	2007–2010	Nationwide	Jan-Apr	1.1 (1.05–1.05)	Meat inspection	[71]
Brazil	2010–2015	Nationwide	Jan-Dec	0.6 (0.62–0.62)	Meat inspection	[72]
Brazil	2004	Paraná	Jan-Dec	9.3 (6.57–12.58)	Meat inspection	[73]
Brazil	2010–2011	São Paulo	Oct-Aug	4.8 (4.58–5.04)	Meat inspection	[74]
Brazil	2003–2004	Rio de Janeiro	Jan-Dec	2.3 (2.07–2.45)	Meat inspection	[75]
Brazil	na	Minas Gerais	na	10.6 (7.55–14.40)	Ab-ELISA	[76]
				4.1 (2.28–6.83)	Immunoblot	
Brazil	2004–2008	Paraná	Jan-Dec	2.2 (2.22–2.24)	Meat inspection	[77]
Brazil	1997–2003	Rio de Janeiro	Jan-Dec	2.0 (1.91–1.99)	Meat inspection	[78]
Brazil	2012	São Paulo	Jan-Dec	2.9 (2.83–3.03)	Meat inspection	[79]
		Minas Gerais		1.8 (1.71–1.93)		
		Goias		0.7 (0.61–0.82)		
		Mato Grosso do Sul		1.1 (0.77–1.58)		
Brazil	2013–2014	Mato Grosso	Jan-Dec	0.1 (0.09–0.09)	Meat inspection	[80]
Brazil	2009	Minas Gerais	na	2.5 (0.92–5.36)	Ab-ELISA	[81]
Brazil	2009–2010	Bahia	Jan-Jan	3.6 (3.43–3.69)	Meat inspection	[82]
Brazil	2008	Goiás	Jan-Dec	0.7 (0.67–0.73)	Meat inspection	[83]
Brazil	2000	Parana	Jul-Dec	3.8 (3.60–4.07)	Meat inspection	[84]
Brazil	2009–2013	Rio Grande do Sul	Jan-Dec	2.5 (2.28–2.78)	Meat inspection	[85]
Brazil	2011–2013	Rio Grande do Sul & Tocantins	Jan-Apr	5.0 (2.80–8.03)	Meat inspection	[86]
Brazil	na	Paraíba	na	2.7 (2.11–3.46)	Ab-ELISA	[87]
Canada	1992	Ontario	Oct; Dec	5.8 (4.81–6.82)	Meat inspection	[88]
Canada	2000	Alberta	Apr	7.9 (4.02–13.72)	Meat inspection	[89]
Ecuador	2000–2001	Imbabura; Pichincha	Dec-Jan	5.8 (3.71–8.57)	Ag-ELISA	[47]
				0.3 (0.01–1.48)	Meat inspection	
				2.5 (1.32–4.40)	Ag-ELISA	
				0.5 (0.06–1.66)	Meat inspection	
Mexico	2008–2009	Nationwide	Oct-Jul	0.2 (0.17–0.25)	Meat inspection	[90]
USA	1992–1993	Idaho	May-May	8.9 (8.09–9.66)	Meat inspection	[91]

*Abbreviations*: *CI* confidence interval, *na* information not provided

^a^First-level administration

**Fig. 4:**
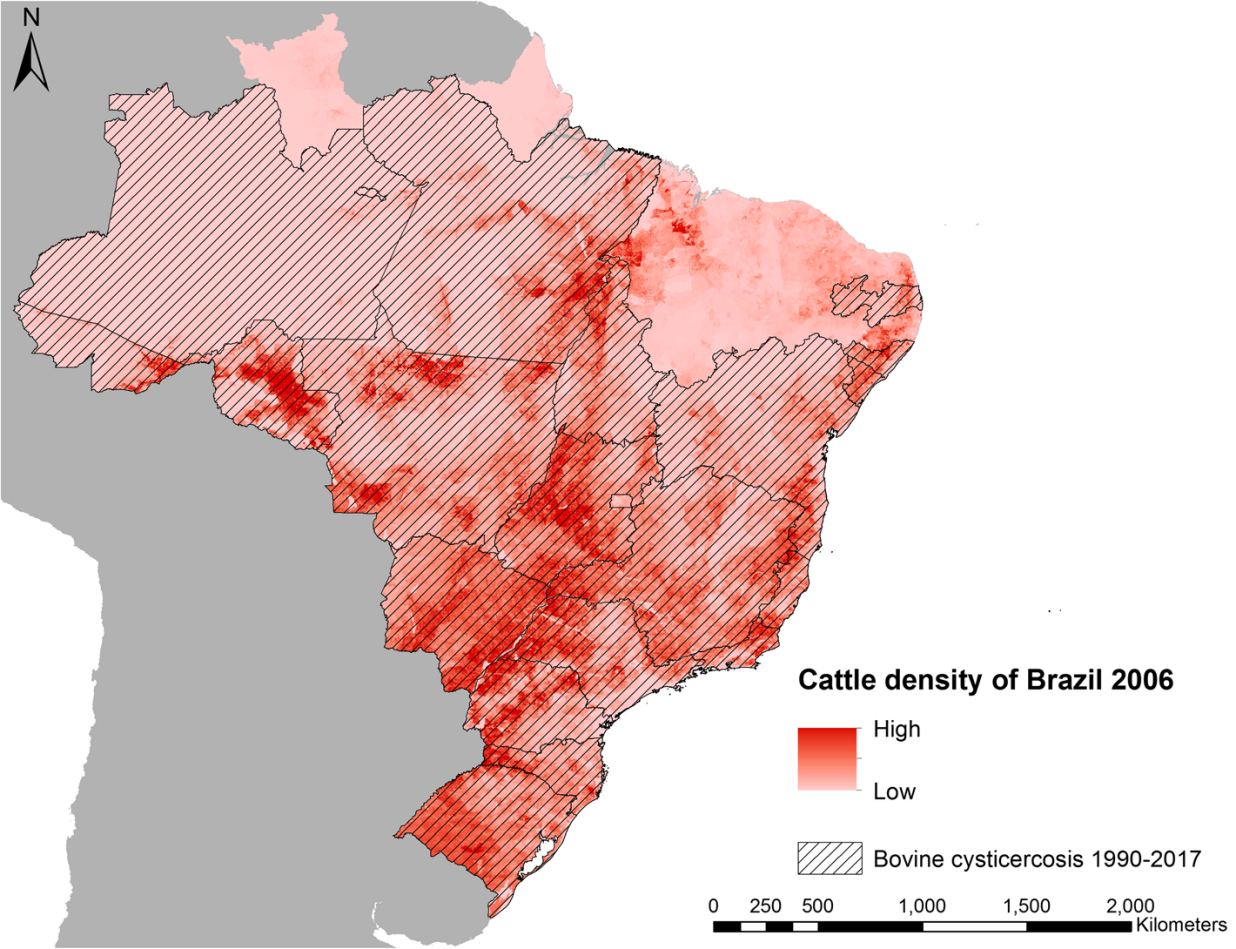
States in Brazil with bovine cysticercosis during 1990–2017 and the modelled cattle density for Brazil in 2006 [12]

Brazil was the only country, where data on bovine cysticercosis could be obtained from several regions of the country. In the period 1990–2017, bovine cysticercosis was reported in 70% (19/27) of the states (first-level administration) in Brazil. As an illustration of risk of bovine cysticercosis, Fig. 4 shows the 2006 modelled density of cattle in Brazil [12], overlaid with the 19 states from where bovine cysticercosis was reported during 1990–2017.

## Discussion

This review shows that *T. saginata* is widely distributed across the Americas. Taeniosis was widely reported, but not always at the species level. The estimation of the distribution and the prevalence of taeniosis is severely complicated by the lack of specific symptoms in tapeworm carriers [2], and the fact that the disease is not commonly notifiable. Some studies differentiated between *T. saginata* taeniosis and the much more dangerous infection, *T. solium* taeniosis. This was usually done in studies with a research objective where the study outcome was dependent on the species differentiation. In health care and routine screening, this information is seen to be of less importance to the physicians attending as the anthelminthic treatments prescribed to patients will be effective against either parasite species [13, 14], despite the risk of cysticercosis transmission to either the carrier or their family members if *T. solium* is present.

The majority of the taeniosis infections reported had been identified by microscopic examination for the presence of *Taenia* spp. eggs, which has low sensitivity [15], and cannot be used for determining the diagnosis to species level. Prevalence of taeniosis ranged from very low levels to almost 9%, which is very similar to previous reports of taeniosis prevalence (0.01–10%) from Europe [16]. However, a direct comparison between studies is not appropriate, as variables such as study duration, recruitment criteria, diagnostic methods and standards of randomisation differ across studies. Clinical investigators should be encouraged to adopt a consensus protocol for collecting and analysing data for apparent taeniosis prevalence estimation, which would make comparison between studies and areas less biased.

It is clear from this review that bovine cysticercosis is widely distributed across the mainland of the Americas. It also indicates, however, a scarcity of recent data from the region. *Taenia saginata* taeniosis was reported from both Guatemala and Peru, but we were unable to identify any reports of bovine cysticercosis from either country. More than half of countries found to have bovine cysticercosis were found through the OIE databases [10, 11]. However, bovine cysticercosis is no longer notifiable to the OIE, and the reporting, if any, is not standardised across the countries in the region. The occurrence of bovine cysticercosis could only be georeferenced to a first-level administration in five countries. This illustrates that for most of the countries where *T. saginata* is endemic, more data are needed to pinpoint areas of risk and areas with high transmission rates. Presence of bovine cysticercosis seems to be related to the number of animals within a farm [17], spatial modelling of livestock density could therefore be considered as a first step in estimating areas of risk, such as previously done for *T. solium* [18]. Detailed mapping studies with prevalence of bovine cysticercosis are warranted for all endemic countries; however, such studies only seem to have been performed in Brazil. Bovine cysticercosis prevalence based on meat inspection ranged from very low levels to almost 19%, which is a higher range than recent reports from Europe (< 5%) [8], the Middle East (3%) [19] and Africa (< 4%) [20, 21].

Only five countries on the mainland (Belize, French Guiana, Guyana, Suriname and Panama) did not have reports of taeniosis or bovine cysticercosis. All five countries have cattle industries and in 2016 had an estimated number of cattle: 110,024 in Belize, 18,945 in French Guiana, 10,115 in Guyana, 36,138 in Suriname, and 1,554,200 in Panama [22]. Due to reports of bovine cysticercosis from neighbouring countries, the missing reports could be a result of underreporting rather than absence of the parasite in these populations. Epidemiological surveys should be performed in these five countries in order to confirm or refute the absence of *T. saginata*. The lack of reports of *T. saginata* from the smaller Caribbean islands, except for one case from the US Virgin Islands in 1994 [23], could suggest that infection pressure is insufficient to sustain transmission on these islands and reports are a result of small self-limiting outbreaks resulting from imported taeniosis cases. Cattle populations are relatively small on the Caribbean islands with many islands slaughtering less than 1000 cattle annually [22], which would likely mitigate any potential outbreak to burn out quickly. However, bovine cysticercosis was found on the two largest Caribbean islands (Cuba and Hispaniola). On Hispaniola, bovine cysticercosis was only reported in Haiti (Fig. 4); thus, investigations to explore the situation in the Dominican Republic are highly warranted.

There are clear diagnostic issues in terms of both taeniosis and bovine cysticercosis. The sensitivity of meat inspection for bovine cysticercosis can be increased to some extent by making more incisions into organs and muscles of the carcass. However, the risk of contaminating the carcass with microbes that pose a risk to food safety is correlated with the number of incisions made [24]. Since the health risk in *T. saginata* infections are minimal, compared to those of bacterial pathogens for example, an increase of the number of incisions might be unwise or should perhaps be completely avoided under certain circumstances. However, more incisions in the heart has been shown to increase the sensitivity for the diagnosis of bovine cysticercosis compared to the EU-approved routine meat inspection [25]. Still, more research is required to determine whether meat inspection procedures should be changed and what consequences it will have for areas of low and high bovine cysticercosis endemicity, respectively. Another approach to reduce transmission risk could be risk-based meat inspection in countries where appropriate herd level data is obtainable [26].

*Taenia saginata* is prevalent in countries where sanitation standards are high [8], suggesting that elimination of this parasite is extremely difficult. Epidemiological surveys and mapping studies should be performed in all endemic countries in order to evaluate the distribution and the economic burden of *T. saginata* and to assess whether cost-effective intervention measures can be implemented. Standard goals should be put forward in terms of describing a protocol for estimating the distribution, prevalence, incidence and the economic burden of bovine cysticercosis. Based on the economic analyses, cost-effective measures can then be implemented, if appropriate, in the efforts to control *T. saginata*.

## Conclusions

*Taenia saginata* is widely distributed across the Americas, but the epidemiological data available are insufficient to estimate the subnational spatial distribution, prevalence, incidence and intensity of infections. This needs to be addressed through active surveillance and disease detection programmes. Such programmes would ameliorate the lack of data needed to quantify the economic burden bovine cysticercosis imposes on the region. These issues should be addressed in order to assess the need and advocate for implementation of cost-effective control measures against *T. saginata* if necessary.

## Additional files

Additional file 1: Review protocol and PRISMA checklist. (PDF 527 kb)

Additional file 2: **Table S1.** Identified literature supporting the presence of *Taenia saginata* in the Americas during 1990-2017. **Table S2.** States in Brazil with bovine cysticercosis in 1990–2017. **Table S3.** Countries in the Americas with *Taenia saginata* occurrence in 1990-2017. (XLSX 37 kb)

## Abbreviation

CI: Confidence interval
