# Supplementary material for: Epidemiology of Taenia saginata taeniosis/cysticercosis: a systematic review of the distribution in the Americas
Source: Parasit Vectors. 2018 Sep 20;11:518. doi: 10.1186/s13071-018-3079-y (PMC6149206; doi:10.1186/s13071-018-3079-y)
Supplement: Supplementary file 1 — Review protocol and PRISMA checklist. (PDF 527 kb) [file 13071_2018_3079_MOESM1_ESM.pdf]

Distribution of *Taenia Saginata* in the Americas - Systematic Review

Aim: To synthesis the current evidence on presence/absence and prevalence (where reported) of *T. saginata* (taeniosis/cysticercosis) in the Americas

Questions to answer:

Which countries have reported the presence of *T. saginata* in cattle and/or humans since 1990?

For which countries are prevalence data available and what is the quality of that data?

Are specific geographical locations available for these data?

Methods:

The review will be conducted in line with the PRIMSA statement 2009 (http://www.bmj.com/content/339/bmj.b2700#alternate) and will include each item on the PRIMSA checklist http://www.prisma-statement.org.

Articles will be selected for inclusion into the systematic review through the identification of all potentially relevant citations through the search strategy. The citations within identified articles will also be included in the screening process. Duplicates will be excluded, followed by screening of titles and abstracts with articles excluded if they do not explicitly report occurrence or prevalence of *T. saginata*. Full text articles will then be screened for exclusion criteria with those remaining utilised in the review.

Full text articles will then be read and relevant data extracted and entered into a Microsoft excel spreadsheet.

Databases;

- Pubmed (http://www.ncbi.nlm.nih.gov/pubmed)
- Web of Science (http://ipscience.thomsonreuters.com/product/web-of-science/)
- OpenGrey (http://www.opengrey.eu/)
- CABDirect (http://www.cabdirect.org/)

Search term (Pubmed): (cysticerc* OR cisticerc* OR "C. bovis" OR taenia* OR tenia* OR saginata OR taeniosis OR teniosis OR taeniasis OR ténia OR taeniid OR cysticerque OR Taeniarhynchus) AND (America OR USA OR Brazil OR Argentina OR Canada OR Peru OR Chile OR Ecuador OR Bolivia OR Paraguay OR Costa Rica OR Uruguay OR Bermuda OR Greenland OR Caribbean Netherlands OR Saint Barts OR Saint Pierre and Miquelon OR Falkland Islands OR Anguilla OR Antigua and Barbuda OR Aruba OR Bahamas OR Barbados OR Belize OR Bonaire OR British Virgin Islands OR Bermuda OR Cayman Islands OR Colombia OR Costa Rica OR Cuba OR Curaçao OR Dominica OR Dominican Republic OR El Salvador OR French Guiana OR Grenada OR Guadeloupe OR Guatemala

OR Guyana OR Haiti OR Honduras OR Jamaica OR Martinique OR Mexico OR Montserrat OR Netherlands Antilles OR Nicaragua OR Panama OR Puerto Rico OR Saba OR Saint Kitts and Nevis OR Saint Lucia OR Saint Vincent and the Grenadines OR Saint Eustatius OR Sint Maarten OR Saint Martin OR Suriname OR Trinidad and Tobago OR Turks and Caicos Islands OR US Virgin Islands OR Venezuela)

Inclusion/exclusion:

- **Exclusion criteria:**
  - studies concerning a different parasite than *T. saginata*
  - studies reporting data from outside authors countries of responsibility
  - studies reporting/using data older than 1990 or published after December 31st 2017
  - studies reporting results out of the scope of the review questions
  - duplicated data

- **Languages:** All

- **Year data collection:** 1st January 1990 – 31st December 2017

- **Geographical range:** All countries/territories within the Americas

PRISMA 2009 Checklist

Section/topic	#	Checklist item	Reported on page #
**TITLE**			
Title	1	Identify the report as a systematic review, meta-analysis, or both.	1
**ABSTRACT**			
Structured summary	2	Provide a structured summary including, as applicable: background; objectives; data sources; study eligibility criteria, participants, and interventions; study appraisal and synthesis methods; results; limitations; conclusions and implications of key findings; systematic review registration number.	2
**INTRODUCTION**			
Rationale	3	Describe the rationale for the review in the context of what is already known.	2-3
Objectives	4	Provide an explicit statement of questions being addressed with reference to participants, interventions, comparisons, outcomes, and study design (PICOS).	NA
**METHODS**			
Protocol and registration	5	Indicate if a review protocol exists, if and where it can be accessed (e.g., Web address), and, if available, provide registration information including registration number.	3
Eligibility criteria	6	Specify study characteristics (e.g., PICOS, length of follow-up) and report characteristics (e.g., years considered, language, publication status) used as criteria for eligibility, giving rationale.	3-4
Information sources	7	Describe all information sources (e.g., databases with dates of coverage, contact with study authors to identify additional studies) in the search and date last searched.	3-4
Search	8	Present full electronic search strategy for at least one database, including any limits used, such that it could be repeated.	4
Study selection	9	State the process for selecting studies (i.e., screening, eligibility, included in systematic review, and, if applicable, included in the meta-analysis).	3-4
Data collection process	10	Describe method of data extraction from reports (e.g., piloted forms, independently, in duplicate) and any processes for obtaining and confirming data from investigators.	4
Data items	11	List and define all variables for which data were sought (e.g., PICOS, funding sources) and any assumptions and simplifications made.	4
Risk of bias in individual studies	12	Describe methods used for assessing risk of bias of individual studies (including specification of whether this was done at the study or outcome level), and how this information is to be used in any data synthesis.	NA
Summary measures	13	State the principal summary measures (e.g., risk ratio, difference in means).	NA
Synthesis of results	14	Describe the methods of handling data and combining results of studies, if done, including measures of consistency (e.g., I^2) for each meta-analysis.	NA

PRISMA 2009 Checklist

Section/topic	#	Checklist item	Reported on page #
Risk of bias across studies	15	Specify any assessment of risk of bias that may affect the cumulative evidence (e.g., publication bias, selective reporting within studies).	NA
Additional analyses	16	Describe methods of additional analyses (e.g., sensitivity or subgroup analyses, meta-regression), if done, indicating which were pre-specified.	NA
**RESULTS**			
Study selection	17	Give numbers of studies screened, assessed for eligibility, and included in the review, with reasons for exclusions at each stage, ideally with a flow diagram.	5
Study characteristics	18	For each study, present characteristics for which data were extracted (e.g., study size, PICOS, follow-up period) and provide the citations.	NA
Risk of bias within studies	19	Present data on risk of bias of each study and, if available, any outcome level assessment (see item 12).	NA
Results of individual studies	20	For all outcomes considered (benefits or harms), present, for each study: (a) simple summary data for each intervention group (b) effect estimates and confidence intervals, ideally with a forest plot.	NA
Synthesis of results	21	Present results of each meta-analysis done, including confidence intervals and measures of consistency.	NA
Risk of bias across studies	22	Present results of any assessment of risk of bias across studies (see Item 15).	NA
Additional analysis	23	Give results of additional analyses, if done (e.g., sensitivity or subgroup analyses, meta-regression [see Item 16]).	NA
**DISCUSSION**			
Summary of evidence	24	Summarize the main findings including the strength of evidence for each main outcome; consider their relevance to key groups (e.g., healthcare providers, users, and policy makers).	13
Limitations	25	Discuss limitations at study and outcome level (e.g., risk of bias), and at review-level (e.g., incomplete retrieval of identified research, reporting bias).	12
Conclusions	26	Provide a general interpretation of the results in the context of other evidence, and implications for future research.	11-13
**FUNDING**			
Funding	27	Describe sources of funding for the systematic review and other support (e.g., supply of data); role of funders for the systematic review.	NA

From: Moher D, Liberati A, Tetzlaff J, Altman DG, The PRISMA Group (2009). Preferred Reporting Items for Systematic Reviews and Meta-Analyses: The PRISMA Statement. PLoS Med 6(7): e1000097. doi:10.1371/journal.pmed1000097

For more information, visit: **www.prisma-statement.org**.
